# SciReader enables reading of medical content with instantaneous definitions

**DOI:** 10.1186/1472-6947-11-4

**Published:** 2011-01-25

**Authors:** Patrick R Gradie, Megan Litster, Rinu Thomas, Jay Vyas, Martin R Schiller

**Affiliations:** 1 School of Life Sciences, University of Nevada, Las Vegas, 4505 Maryland Pkwy., Las Vegas, NV 89154-4004 USA; 2 Department of Molecular, Microbial, and Structural Biology, University of Connecticut Heath Center, 263 Farmington Ave., Farmington, CT 06030-3305 USA

## Abstract

**Background:**

A major problem patients encounter when reading about health related issues is document interpretation, which limits reading comprehension and therefore negatively impacts health care. Currently, searching for medical definitions from an external source is time consuming, distracting, and negatively impacts reading comprehension and memory of the material.

**Methods:**

*SciReader *was built as a Java application with a Flex-based front-end client. The dictionary used by *SciReader *was built by consolidating data from several sources and generating new definitions with a standardized syntax. The application was evaluated by measuring the percentage of words defined in different documents. A survey was used to test the perceived effect of SciReader on reading time and comprehension.

**Results:**

We present *SciReader*, a web-application that simplifies document interpretation by allowing users to instantaneously view medical, English, and scientific definitions as they read any document. This tool reveals the definitions of any selected word in a small frame at the top of the application. *SciReader *relies on a dictionary of ~750,000 unique Biomedical and English word definitions. Evaluation of the application shows that it maps ~98% of words in several different types of documents and that most users tested in a survey indicate that the application decreases reading time and increases comprehension.

**Conclusions:**

*SciReader *is a web application useful for reading medical and scientific documents. The program makes jargon-laden content more accessible to patients, educators, health care professionals, and the general public.

## Background

While 99% of people in the United States are considered literate, current estimates indicate that only 17% - 28% have a basic science literacy and only about 150 million people are what doctors consider medically literate [1-4]. Low scientific and medical literacy renders medical documentation difficult to read and impacts the health care system. Studies link low medical literacy to poor health status, lower self-reporting of medical conditions, lower compliance with doctor's directions, increased rates of hospitalization, and increased health care costs [1]. Medical literacy is partially hindered by the large medical vocabulary, which far exceeds the knowledge boundaries of most people.

Major initiatives in the United States have yielded a modest increase in literacy by about 15% since the 1980s [1-4]. However, the average American is still not considered scientifically literate. There are now several types of tools that facilitate literacy. Web browsers provide access to millions of documents by anyone with internet access and digital document readers focus on user-friendly presentation of many types of documents. One remaining limitation is the problem of document interpretation. This is true especially in health care where the highly technical terminology often obscures comprehension, and limits understanding to all but a small group of experts.

Readers tend to invoke three general strategies while reading a jargon-rich medical or scientific document. First, the reader may opt to ignore the unknown word altogether. Although this may decrease reading time, it by no means aids in understanding. The second strategy is to infer the meaning of the unknown word from the surrounding text, which is an inexact and error-prone approach. Finally, a person may decide to consult an outside source such as a dictionary. This strategy tends to be time consuming and can negatively impact reading comprehension and memory of the material [5].

A literary tool that simplifies interpretation would make jargon-laden content more accessible to patients, educators, health care professionals, as well as the general public. To address this problem, we have built *SciReader*, a open access web-application that allows users to instantaneously view English, medical, and scientific word definitions as they read any document. This tool reveals the definitions of any selected word in small frame at the top of the application.

## Implementation

### Application and Database Design

**Figure 1:**
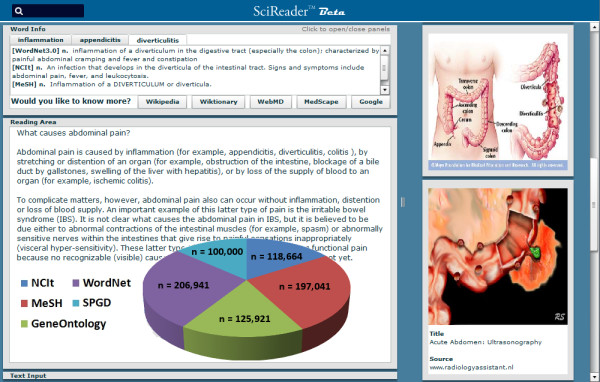
**SciReader vocabulary and user interface**. Image of the *SciReader *user interface. The top frame displays definitions that are revealed when any word ("diverticulitis" in this case) is selected in the bottom document window. The vocabulary search bar is signified by a magnifying glass. The right frame shows results retrieved from a Google image search for the selected word. A overlaid Pie graph shows the number of unique definitions in sources of the *SciReader *vocabulary. NCIt = National Cancer Institute thesaurus, MeSH = Medical Subject Headings, GO = Gene Ontology; SPGD = standardized protein and gene definitions.

The *SciReader *web server was coded in Java with a Flex-based front end client, which requires the commonly used Flash plug-in. Our initial implementation of *SciReader *relies on a dictionary of ~750,000 unique Medical, Biological, and English word definitions. The vocabulary was derived by consolidating data from several sources including WordNet [6], Open Biomedical Ontologies [7], NCI thesaurus [8], Medical Subject Headings [9], and the Gene Ontology [10]. Additional word definitions for numerous protein and genes in RefSeq were generated in a standardized syntax using functions from the Gene Ontology [10,11]. These vocabularies were implemented in a MySQL database. The number of word definitions in these sources is shown in Figure 1. *SciReader *can be readily extended to include scientific vocabularies from any other field.

### Word Search Algorithm

When a user clicks a word in the "Reading Area" the following occurs:

1. Both the clicked word and the sentence the word belongs to are sent to the server.

2. The server then creates a list of possible word phrases by performing a database search for the following words:

a. The selected word.

b. Words that end with the selected word.

c. Words that begin with the selected word.

*In order to cull the results returned, when possible, the server will use both the selected word and a word to the right and/or left.

3. Once this set of words is found the longest word phrase length is determined by selecting the longest word phrase length of all the returned word phrases (the word phrase length is returned with the database search for each word phrase).

4. Using the longest word length, a set of word phrases is generated from the sentence by creating all possible word phrases that are at most the length of the longest word phrase length returned from #3.

5. Each word generated from #4 is then matched against the possible word list generated from #2 and the definitions for each matching word are sent back to the client user.

6. The definitions for each word phrase found in the sentence are shown to the user.

### Client Trial

In order to gauge the effectiveness and usability of *SciReader*, 105 students in a introductory college biology class (Biology 100 at UNLV) were provided access to the *SciReader *application and asked to answer a couple of survey questions. The subjects were provided access for ~1 month to seven chapters in their biology textbook in the *SciReader *application. Two different survey questions were related to reading comprehension and reading time. Students were asked to respond to the following statements: "I think that using *SciReader *while reading my science textbook decreased the time it took for me to read." and "I think that using *SciReader *improved my understanding of the material I read.". Students selected responses from a 5-point Likert scale with 1 = strongly agree, 2 = agree, 3 = neutral, 4 = disagree, 5 = strongly disagree. An average score was calculated and used as a metric to measure the perceived effectiveness of *SciReader*. It is important to note that a lower score correspond to a readers agreement with the statement. The survey protocol was approved by the UNLV Social/Behavioural Institutional Review Board (IRB protocol number: 1007-3529M).

## Results

### Application Design

The view of the *SciReader *user interface shown in Figure 1 contains a small dynamic window frame that displays multiple definitions and a window showing the uploaded text. The application accepts text input from a third window that disappears after text is loaded. Definitions are displayed when any word is selected with a mouse click.

*SciReader *has a number of basic features that facilitate ease-of-use. In addition to single word definitions, *SciReader *scans sentences to identify compound word phrases. When a word is selected, multiple definitions are returned with their database source and associated part of speech, if known. Importantly, for reading high-level content, the definitions of words within the definition window can be identified by selecting the word. Since many definitions may still be too complicated for users with poor literacy, *SciReader *provides links to articles about a selected word from Wikipedia, Wiktionary, WebMD, MedScape, Google, and The Free Dictionary. Furthermore, a link to images for the word is also accessed through the application. These links provide additional depth should the definitions provided prove insufficient for comprehension. The application search bar can also be used as a medical or biological dictionary to retrieve the definition of individual words.

### Database Word Mapping Efficiency

To determine the efficiency of word mapping in *SciReader *we loaded several scientific documents of similar length written for readers with varying levels of expertise. Results from analysis of a typical newspaper article, college level textbook, and biological journal article demonstrated that 98 ± 1% (n = 3) of all words are mapped with at least one definition; proper nouns were not included in this calculation (Table 1). To facilitate construction of a more comprehensive vocabulary, when a client selects a word for which there is no definition in the *SciReader *database, the word is recorded to a database table so that definitions can be added in the future.

**Table 1:** Evaluation of word mapping in *SciReader*

Document	# words	# word with definitions	Percent with definitions
Judge Invalidates Human Gene Patent., J. Schwartz and A Pollack, The New York Times March 29, 2010.	772	768	100

WormBook, The Online Review of C. elegans Biology., The *C. elegans *M. Chalfie and Research Community, editors, Pasadena (CA): WormBook; 2005, Chapter 5.1.	761	744	98

Vyas J, Gryk MR, and Schiller. (2009). Venn, a tool for titrating sequence conservation onto protein structures. Nucleic Acids Res. 37, e124. (results section) [23]	800	781	98

**Average**	**778**	**764**	**98 ± 1**

### Survey

We surveyed 105 college students to determine if *SciReader *helped address problems in science/medical literacy (Table 2). Entry-level college students were chosen because these individuals have only been exposed to a high-school level of biology vocabulary. The students were provided access to seven chapters in an introductory level college biology text book and then asked to respond to whether *SciReader *helped reduce reading time or increased reading comprehension; two problems that are associated with poor scientific literacy [2]. Student selected their responses from a 5-point Likert scale indicating different levels of agreement with the statements provided (Table 2). Scores for the survey data are provided in Additional file 1. The opinion of *SciReader *users showed an average score of 2.4 with 50% of users indicating that the application reduced the time needed to read the chapters. A score of 2.0 was observed for increased reading comprehension where 76% of users thought that the application helped them better understand the chapter. These results indicate that *SciReader *is a tool that is perceived to be beneficial for reading technical content by the majority of users.

**Table 2:** Survey responses to *SciReader*

Question	Strongly Agree (1)	Agree (2)	Neutral (3)	Disagree (4)	Strongly Disagree (5)	Average Opinion
Decreased reading time	18.1%	32.4%	37.1%	11.4%	1.0%	2.4

Increased comprehension	23.8%	52.4%	21.9%	1.0%	1.0%	2.0

This table summarizes the responses of 105 individuals with a high school education.

The 5-point Likert scale is described in methods, scores are indicated in parentheses.

## Discussion

Low medical and scientific literacy is a longstanding problem dating back to the late 1950s [12]. Most publications in these fields are focused upon identifying the problem [13-17], measuring literacy [18,19], and assessing its impact on health care or education [20,21]. However, reports on progress toward improving literacy are generally limited. One example is the Medline Plus Kiosk, a community outreach project aimed at increasing medical literacy by presenting people with easy to comprehend medical information [22]. To further medical literacy we report the construction of *SciReader*, a new computational tool that can be used synergistically with internet applications. *SciReader *allows people to read medical content and obtain word definitions in the same view as the document being read.

*SciReader *is a unique tool that automates the tedious process of searching for, and evaluating scientific and medical terminology during the reading process. *SciReader *integrates a number of important text-based functions found in existing online dictionaries and ontologies, as well as search engines. A number of dictionaries and ontologies, which currently exist as separate sources are now accessible in a single search through the search engine embedded in *SciReader*. Typical content searches for images and detailed articles, normally performed with a search engine, are now coupled to selection of any word in *SciReader*. *SciReader *returns a series of related images from a Google search and also loads links to the Wikipedia encyclopedia and to articles from the WebMD and Medscape knowledgebases.

All of these functions can be accomplished without *SciReader*; however, integrating these tools into a unified view may have distinct advantages not realized in the separate applications by themselves. The recondite nature of scientific and medical content requires many readers to repetitively shift their train of thought and research the meanings of words. Not only is this a deterrent, but also negatively impacts, reading time, comprehension, and memory of the material read [5]. *SciReader *provides on the spot definitions and images for most words in a medical document. Even if the definition provided by *SciReader *does not help the reader, the search retrieves the images and links that a reader would normally pursue in the next attempts to ascertain comprehension.

One limitation in *SciReader *is that some of the definitions may be too complicated for a person with poor literacy to understand. In this situation, where more information is required, links to a WebMD, Wikipedia, or Wiktionary article and images about the topic are provided. Alternatively, a user can use Google. While these are not perfect solutions, they will facilitate learning more about the unknown word. Nevertheless, *SciReader *is a computational reading tool that can be used in conjunction with other web tools to promote medical/scientific literacy.

## Conclusions

In summary, *SciReader *can be used to assist with interpreting medical documents for medical professionals and non-experts such as medical students, patients, and the general public. The application has the potential to improve health care by increasing their comprehension of medical and/or scientific literature so that patients can better understand their ailments and treatments.

## Availability and requirements

• **Project name**: SciReader

• **Project home page**: http://scireader.bio-toolkit.com

• **Operating system**: Platform independent

• **Programming Language**: Java, Flex

• **Other requirements**: Flash plug-in

• **License**: Free for academic use

• **Any restriction to use by non-academics**: License required

## Competing interests

The Board of Regents of the Nevada System of Higher Education, on behalf of the University of Nevada, Las Vegas, has filed a patent application that is pending. None of the authors have received compensation in any form that would be considered a competing interest nor is there any current plan to develop SciReader into a business. SciReader has not been licensed to any commercial company or government entity.

## Authors' contributions

The application was designed and built by PG. Analysis was by PG and MS. MS prepared the manuscript. JV, RT, PG built the database. ML conducted the survey. MS conceptualized the study. All authors have read and approved the final manuscript.

## Pre-publication history

The pre-publication history for this paper can be accessed here:

http://www.biomedcentral.com/1472-6947/11/4/prepub

## Supplementary Material

Additional File 1 **excel, SciReader Survey Data, This excel file contains the 5-point Likert scale data for the students surveyed**. Click here for file

## References

[B1] Andrus MR Roth MT Health literacy: a review Pharmacotherapy 2002 22 282 302 10.1592/phco.22.5.282.33191 11898888

[B2] Gross L Scientific illiteracy and the partisan takeover of biology PLoS Biol 2006 4 e167 10.1371/journal.pbio.0040167 16605305 PMC1436033

[B3] Scientific Literacy: How Do Americans Stack Up? http://www.sciencedaily.com/releases/2007/02/070218134322.htm

[B4] Human Development Report 2009 2009 Palgrave Macmillian. New York

[B5] Knight S Dictionary use while reading: the effects on comprehension and vocabulary acquisition for students of different verbal abilities Mod. Lang. J 1994 285 289 10.2307/330108

[B6] Sigman M Cecchi GA Global organization of the Wordnet lexicon Proc. Natl. Acad. Sci. USA 2002 99 1742 1747 10.1073/pnas.022341799 PMC122261 11830677

[B7] Smith B Ashburner M Rosse C Bard J Bug W Ceusters W Goldberg L Eilbeck K Ireland A Mungall C Leontis N Rocca-Serra P Ruttenberg A Sansone S Scheuermann R Shah N Whetzel P Lewis S The OBO Foundry: coordinated evolution of ontologies to support biomedical data integration Nature Biotech 2007 25 1251 1255 10.1038/nbt1346 PMC2814061 17989687

[B8] Fragoso G de Coronado S Haber M Hartel F Wright L Overview and utilization of the NCI thesaurus Comp. Funct. Genomics 2004 5 648 654 10.1002/cfg.445 18629178 PMC2447470

[B9] Lipscomb CE Medical Subject Headings (MeSH) Bull Med Libr Assoc 2000 88 265 266 10928714 PMC35238

[B10] Ashburner M Ball C Blake J Botstein D Butler H Cherry J Davis A Dolinski K Dwight S Eppig J Harris M Hill D Issel-Tarver L Kasarskis A Lewis S Matese J Richardson J Ringwald M Rubin G Sherlock G Gene Ontology: tool for the unification of biology Nat.Genet 2000 25 25 29 10.1038/75556 10802651 PMC3037419

[B11] Sayers EW Barrett T Benson DA Bolton E Bryant SH Canese K Chetvernin V Church DM Dicuccio M Federhen S Feolo M Geer LY Helmberg W Kapustin Y Landsman D Lipman DJ Lu Z Madden TL Madej T Maglott DR Marchler-Bauer A Miller V Mizrachi I Ostell J Panchenko A Pruitt KD Schuler GD Sequeira E Sherry ST Shumway M Sirotkin K Slotta D Souvorov A Starchenko G Tatusova TA Wagner L Wang Y John Wilbur W Yaschenko E Ye J Database resources of the National Center for Biotechnology Information Nucleic Acids Res 2010 38 D5 16 10.1093/nar/gkp967 19910364 PMC2808881

[B12] Laugksch R Scientific Literacy: A Conceptual Overview Science Education 1999 84 71 94 10.1002/(SICI)1098-237X(200001)84:1<71::AID-SCE6>3.0.CO;2-C

[B13] Culliton BJ The Dismal State of Scientific Literacy: Studies find only 6% of Americans and 7% of British meet standard for science literacy Science 1989 243 600 10.1126/science.243.4891.600 17834220

[B14] Snow CE Academic language and the challenge of reading for learning about science Science 2010 328 450 452 10.1126/science.1182597 20413488

[B15] Webb P Science education and literacy: imperatives for the developed and developing world Science 2010 328 448 450 10.1126/science.1182596 20413487

[B16] Pearson PD Moje E Greenleaf C Literacy and science: each in the service of the other Science 2010 328 459 463 10.1126/science.1182595 20413491

[B17] Zarcadoolas C Pleasant A Greer DS Understanding health literacy: an expanded model Health Promot Int 2005 20 195 203 10.1093/heapro/dah609 15788526

[B18] Ashida S Goodman M Pandya C Koehly LM Lachance C Stafford J Kaphingst KA Age Differences in Genetic Knowledge, Health Literacy and Causal Beliefs for Health Conditions Public Health Genomics 2010 in press 20829577 10.1159/000316234 PMC3136390

[B19] McCormack L Bann C Squiers L Berkman ND Squire C Schillinger D Ohene-Frempong J Hibbard J Measuring health literacy: a pilot study of a new skills-based instrument J Health Commun 2010 15 Suppl 2 51 71 10.1080/10810730.2010.499987 20845193 PMC12086698

[B20] Garcia SF Hahn EA Jacobs EA Addressing low literacy and health literacy in clinical oncology practice J Support Oncol 2010 8 64 69 20464884 PMC3127453

[B21] Paasche-Orlow MK Wolf MS Promoting health literacy research to reduce health disparities J Health Commun 2010 15 Suppl 2 34 41 10.1080/10810730.2010.499994 20845191

[B22] Teolis MG A MedlinePlus Kiosk Promoting Health Literacy J Consum Health Internet 2010 14 126 137 10.1080/15398281003780966 20808715 PMC2929770

[B23] Vyas J Gryk MR Schiller MR VENN, a tool for titrating sequence conservation onto protein structures Nucleic Acids Res 2009 37 e124 10.1093/nar/gkp616 19656955 PMC2764419

